# Spontaneous coronary artery dissection in patients with prior psychophysical stress: a systematic review of case reports and case series

**DOI:** 10.1186/s12872-024-03902-2

**Published:** 2024-05-03

**Authors:** Kaveh Hosseini, Parisa Fallahtafti, Payam Roudbari, Hamidreza Soleimani, Negin Abiri Jahromi, Mana Jameie, Yaser Jenab, Ali Moradi, Ali Ajam, Narges Heydari, Toshiki Kuno, Nupoor Narula, Polydoros N. Kampaktsis

**Affiliations:** 1 Cardiac Primary Prevention Research Center, Cardiovascular Disease Research Institute, Tehran University of Medical Sciences, Tehran, 1419733141 Iran; 2 Tehran Heart Center, Cardiovascular Disease Research Institute, Tehran University of Medical Sciences, Tehran, 1419733141 Iran; 3 School of Medicine, Tehran University of Medical Sciences, Tehran, Iran; 4 Non-Communicable Disease Research Center, Endocrinology and Metabolism Population Sciences Institute, Tehran University of Medical Sciences, Tehran, 1411713139 Iran; 5 Department of Medicine and Vascular Medicine Institute, University of Pittsburgh School of Medicine and UPMC, Pittsburgh, USA; 6 Faculty of Medicine, Isfahan University of Medical Sciences, Isfahan, Iran; 7 Department of Medicine, Montefiore Medical Center, New York, NY 10461 USA; 8 Weill Cornell Medicine, New York Presbyterian, New York City, USA; 9 Division of Cardiology, Columbia University Irving Medical Center, New York, NY 10032 USA

**Keywords:** Spontaneous coronary artery dissection, SCAD, Acute coronary syndrome, Stress

## Abstract

**Background:**

Spontaneous coronary artery dissection (SCAD) is an underdiagnosed cause of acute coronary syndrome, particularly in younger women. Due to limited information about SCAD, case reports and case series can provide valuable insights into its features and management. This study aimed to comprehensively evaluate the features of SCAD patients who experienced psychophysical stress before the SCAD event.

**Methods:**

We conducted an electronic search of PubMed, Scopus, and Web of Science from inception until January 7, 2023. We included case reports or series that described patients with SCAD who had experienced psychophysical stress before SCAD. Patients with pregnancy-associated SCAD were excluded from our analysis.

**Results:**

In total, we included 93 case reports or series describing 105 patients with SCAD. The average patient age was 44.29 ± 13.05 years and a total of 44 (41.9%) of patients were male. Among the included SCAD patients the most prevalent comorbidities were fibromuscular dysplasia (FMD) and hypertension with the prevalence of 36.4 and 21.9%, respectively. Preceding physical stress was more frequently reported in men than in women; 38 out of 44 (86.4%) men reported physical stress, while 36 out of 61 (59.1%) females reported physical stress (*p* value = 0.009). On the other hand, the opposite was true for emotional stress (men: 6 (13.6%)), women: 29 (47.6%), *p* value < 0.001). Coronary angiography was the main diagnostic tool. The most frequently involved artery was the left anterior descending (LAD) (62.9%). In our study, recurrence of SCAD due to either the progression of a previous lesion or new SCAD in another coronary location occurred more frequently in those treated conservatively, however the observed difference was not statistically significant (*p* value = 0.138).

**Conclusion:**

While physical stress seems to precede SCAD in most cases, emotional stress is implicated in females more than males.

**Supplementary Information:**

The online version contains supplementary material available at 10.1186/s12872-024-03902-2.

## Introduction

Spontaneous coronary artery dissection (SCAD) is becoming an increasingly recognized etiology of acute coronary syndrome (ACS), cardiac arrest, and sudden cardiac death [1]. SCAD is characterized by a non-atherosclerotic, non-iatrogenic, and non-traumatic spontaneous tear in coronary arteries, which leads to a collapsed arterial lumen (true lumen) due to the formation of an intramural hematoma (IMH) (false lumen), resulting in compromised coronary blood flow and myocardial infarction (MI) [2].

SCAD is responsible for 1–4% of ACS cases in the general population but is estimated to affect as many as 35% of women under 50 [3]. It is one of the leading causes of pregnancy-associated myocardial infarction (PAMI) [4]. Nevertheless, owing to its underdiagnosis and misdiagnosis with atherosclerotic ACS, its actual prevalence is higher than previously reported [5]. Despite this, the etiology of SCAD remains uncertain. There is increasing interest in discovering the main risk factors and triggers behind its pathogenesis. The current knowledge of SCAD has mainly remained limited to single-center case reports and case series data, with only a handful of large-scale cohorts and no randomized clinical trials investigating its pathogenesis, management, and prognosis [6–9]. Thus, exploring case reports and case series regarding SCAD could provide helpful information regarding disease characteristics and management. In addition, case reports and case series can provide insight into uncommon conditions that may be challenging for practitioners to diagnose and treat, offering guidance on how to manage these conditions.

Previous studies have suggested a minor role of traditional cardiovascular risk factors, except hypertension, in SCAD occurrence [2]. Some factors that are believed to contribute to SCAD pathogenesis potentially include underlying arteriopathies (such as fibromuscular dysplasia (FMD)), female sex, pregnancy/other hormonal changes, systemic inflammatory conditions, and connective tissue disorders [10]. One important predisposing factor includes psychophysical stress, and its role in SCAD has been introduced as a research priority and a key question by the American Heart Association [2]. More than half of the patients afflicted with SCAD had experienced emotional (including the demise of a family member or marriage and workplace-related issues) or physical stress (including extreme aerobic or anaerobic physical activities, lifting heavy objects, intense Valsalva or coughing) preceding the presentation. The stress experienced by women was predominantly emotional, while that experienced by men was primarily physical [2, 11].

In light of this information, despite the large existing cohorts of SCAD patients, we included only case series and case reports of SCAD patients because more detailed information is available in these types of studies. This systematic review aimed to exclusively evaluate the characteristics and management of patients experiencing SCAD following psychophysical stress.

## Methods

### Study design and search strategy

A systematic review was performed according to the Preferred Reporting Items for Systematic Reviews and Meta-Analyses (PRISMA) guidelines. We conducted a systematic search in the PubMed, Web of Science, and Scopus databases from inception to January 7, 2023. The following keywords were used: “spontaneous coronary artery dissection” and “SCAD”. No restrictions on publication date or publication status were instituted. It is important to note that the search strategy did not include any terms related to stress because the majority of the articles that met the inclusion criteria described the stressful event rather than using the term ‘stress’.

### Eligibility criteria

Two independent authors (P.R. and P.F.) evaluated studies for eligibility by screening titles and abstracts. Subsequently, the full texts of potentially eligible articles were evaluated according to the inclusion criteria. Disagreements were addressed by joint discussion and consensus of the two authors. Finally, all references of the included articles were comprehensively searched to find articles that might have been missed during the initial screening.

Case reports and case series were included if they described at least one patient who had experienced psychophysical stress preceding the onset of SCAD. In our study, psychophysical stress is defined broadly to encompass both rigorous physical activity and psychological events reported by the patient preceding the occurrence of SCAD. Specifically, any intense physical exertion or notable psychological event self-reported by the patient before the SCAD incident is considered indicative of psychophysical stress. In the case series, we only selected patients with psychophysical stress in the data analysis and excluded other reported patients. Patients presenting with pregnancy-associated SCAD (due to the unique and multifaceted stressors associated with pregnancy, which encompass both psychosocial and physiological aspects) or SCAD in the context of illicit drug use, as well as studies in languages other than English, without sufficient data, or with low quality (based on quality assessment method), were excluded.

### Data extraction

Data were independently extracted by two authors (P.R. and P.F.). A third author verified the accuracy of data extraction and addressed any contradictions. Using Microsoft Excel 2019 version (Microsoft Corporation, Redmond, WA, USA), the following data categories were recorded: a) study-related characteristics (first author’s name, publication year, region of the study (according to the World Health Origination regions)), b) patient characteristics (age, sex, past medical history, habitual history), c) psychophysical stress-related characteristics (type, description, time interval with the SCAD occurrence), and d) SCAD-related characteristics (signs and symptoms, clinical diagnosis, electrocardiogram (ECG) findings, dissected artery characteristics, diagnostic and therapeutic approaches, and follow-up events).

### Quality appraisal

Two independent authors (P.R. and P.F.) assessed the quality of the case report/series. Disagreements were addressed by joint discussion and consensus by the two authors. The method proposed by Mohammad Hassan Murad et al. was used to assess the quality of case reports/series [12]. The methodological quality of the case reports and case series was evaluated using six of the eight questions recommended in the referenced article (see Additional file 1). Scores of 5–6, 4, and 0–3 were considered “good,” “fair,” and “poor” studies in terms of quality, respectively. Articles with a score of less than four were excluded from our study.

### Statistical analysis

Data are reported as mean ± SD and/or median with interquartile range. All statistical analyses were conducted using IBM SPSS Statistics version 27 (IBM Corp., Armonk, NY, USA). Statistical analyses were conducted to investigate the relationships and differences among variables in the dataset. Chi-square and Fisher’s Exact tests were employed to assess associations among categorical variables. Comparisons between groups were performed using appropriate statistical tests such as the Independent Samples t-test for normally distributed variables and the Mann-Whitney U test for non-normally distributed variables. The significance level for all performed tests was *p* value< 0.05.

## Results

### Study selection

**Fig. 1:**
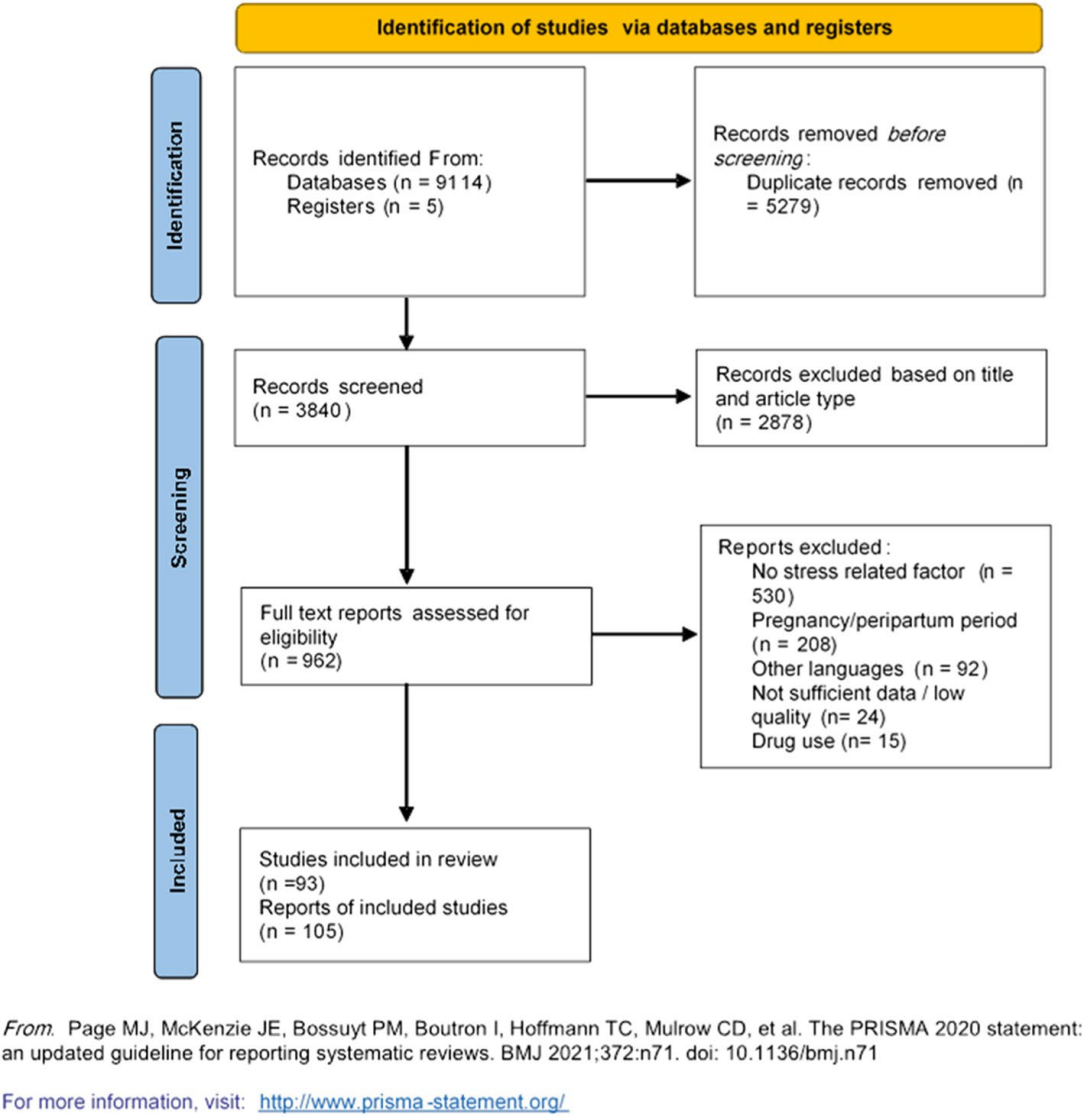
PRISMA 2020 flow diagram for new systematic reviews that included searches of databases and registers only

The PRISMA flow diagram is illustrated in Fig. 1. A total of 9119 articles were retrieved from PubMed, Scopus, and Web of Science. After removing 5279 duplicates using Endnote software version 20.0 (Clarivate PLC, London, United Kingdom), 3840 records were screened based on title and article type, with 2878 being excluded. Of the remaining 962 articles, full-text assessment led to the exclusion of 530 for not reporting stress-related factors prior to SCAD onset, 208 for involving patients in the pregnancy or peripartum period, 92 for being in non-English languages, 24 for low quality, and 15 for reporting illicit drug use. Five studies (eight patients) were added after reviewing the references.

### Study characteristics

A total of 83 included case reports and 10 case series resulted in 105 patients who suffered SCAD following psychophysical stress (83 from case reports and 22 from case series) [13–105]. The largest geographical distribution of these cases was in the Region of the Americas (*n* = 47) and European Region (*n* = 37), followed by the Western Pacific Region (*n* = 16), South–East Asia Region (*n* = 3), Eastern Mediterranean Region (*n* = 2), and African Region (*n* = 1). Most reports were from the United States (*n* = 32), the United Kingdom (*n* = 13), and Canada (*n* = 9).

### Patient characteristics

Patient characteristics are presented in Table 1. The average patient age was 44.29 ± 13.05 years. A total of 44 (41.9%) of patients were male. Female patients were older than their male counterparts (47.15 ± 10.6 vs. 40.32 ± 15.08 years, respectively, *p* value = 0.012).

**Table 1:** Patient characteristics (*N* = 105)

	Total (*N* = 105)	Men (*N* = 44)	Women (*N* = 61)	*P* value
Age (mean ± SD)	44.29 ± 13.05	40.32 ± 15.08	47.15 ± 10.60	0.012
**Psychophysical stress types**				
Physical	70 (66.7%)	38 (86.4%)	32 (52.5%)	0.009
Emotional	31 (29.5%)	6 (13.6%)	25 (41.0%)	< 0.001
Both	4 (3.8%)	0 (0.0%)	4 (6.6%)	0.138
**Past medical history and risk factors**				
Hypertension	23 (21.9%)	7 (15.9%)	16 (26.2%)	0.332
Diabetes	4 (3.8%)	2 (4.5%)	2 (3.3%)	1.000
Dyslipidemia	9 (8.6%)	2 (4.5%)	7 (11.5%)	0.300
Obesity	4 (3.8%)	1 (2.3%)	3 (4.9%)	0.640
CHD	3 (2.9%)	1 (2.3%)	2 (1.6%)	1.000
Heart failure	0 (0.00%)	0 (0.0%)	0 (0.0%)	–
Mental health disorders	12 (11.4%)	2 (4.5%)	10 (16.4%)	0.061
Depression	5 (4.8%)	0 (0.0%)	5 (6.6%)	0.071
Anxiety	5 (4.8%)	0 (0.0%)	5 (6.6%)	0.071
FMD (among 22 evaluated women and 11 evaluated men)	12 (36.4%)	0 (0.0%)	12 (54.5%)	0.001
**Habitual history**				
Smoking	21 (20.00%)	10 (19.2%)	11 (18.0%)	0.62
Alcohol consumption	0 (0.00%)	0 (0.0%)	0 (0.00%)	–

Data are presented as frequency (percentages) or mean ± SD

*CHD* congenital heart defects, *FMD* fibromuscular dysplasia

Hypertension was the most common conventional cardiovascular risk factor (21.9%), followed by dyslipidemia (8.6%), obesity (3.8%), and diabetes mellitus (3.8%). A total of 11.4% of patients had mental health disorders, including depression, anxiety disorder, attention deficit and hyperactivity disorder (ADHD), and posttraumatic stress disorder (PTSD). In terms of congenital anomalies, one patient had an anomalous origin of the coronary artery [58], and two patients had myocardial bridging [60, 95]. Heart failure and alcohol consumption were not reported in any patients. Three patients reported excessive caffeine use before experiencing SCAD [30, 45, 53]. FMD was documented in 12 of 33 patients (36.4%) who were evaluated for this condition [14, 20, 26, 38, 56, 63, 73, 99], none of whom had a known diagnosis of FMD before presenting with SCAD. All of the diagnosed FMD cases were in women. Other documented comorbidities are available in Additional file 2.

### Psychophysical stress

Sole physical and emotional stress were reported among 70 (66.7%) and 31 (29.5%) patients, respectively. Four patients (3.8%) described experiencing both emotional and physical stress. Some of these stressors include aerobic or isometric physical activities, lifting heavy objects, intense Valsalva or coughing, funeral grief, work-related issues, etc. Data for each patient individually are presented in Additional file 2. Physical stress was more frequently seen among men than women; 38 out of 44 (86.4%) men reported physical stress, while 36 out of 61 (59.1%) females reported physical stress (*p* value = 0.009). The converse was true with regard to emotional stress (men: 6 (13.6%), women: 29 (47.6%), *p* value < 0.001). Psychophysical stress was experienced either immediately or for a period before the SCAD event.

### Clinical manifestations

#### Signs and symptoms

The SCAD-related characteristics are presented in Table 2. All patients reported chest pain as their chief complaint except for three [45, 51, 97]. Furthermore, 12 (11.4%), 11 (10.5%), and 9 (8.6%) patients experienced nausea, dyspnea, and diaphoresis, respectively. Less frequent (< 5%) signs and symptoms included vomiting, fatigue, dizziness, and syncope.

**Table 2:** SCAD-related characteristics

	*N* (%)
**ECG (*N*** = 96)^*^	
ST-elevation	55 (57.3%)
ST-depression or T-changes	34 (35.4%)
Normal	7 (7.3%)
**Clinical Presentations**	
Chest pain	102 (97.1%)
Nausea	12 (11.4%)
Dyspnea	11 (10.5%)
Diaphoresis	9 (8.6%)
Cardiogenic shock	5 (4.8%)
Sudden cardiac death	5 (4.8%)
Arrhythmia	7 (6.7%)
**Diagnostic imaging modalities**	
First-line coronary angiography	66 (62.9%)
CCTA-only	3 (2.9%)
CCTA+ complementary coronary angiography	4 (3.8%)
First-line coronary angiography+ IVUS	18 (17.1%)
First-line coronary angiography+ OCT	12 (11.4%)
First-line coronary angiography+ OCT + IVUS	2 (1.9%)
First-line coronary angiography+ CCTA + IVUS	1 (1.0%)
Repeated coronary angiography	
**Number of arteries having SCAD (*N*** **= 93)** †	
Single-vessel SCAD	86 (92.5%)
Two-vessel SCAD	5 (5.4%)
Three-vessel SCAD	2 (2.2%)
**Types of coronary arteries having SCAD (*N*** **= 93)** †	
LMCA	6 (6.5%)
LAD	63 (67.7%)
LCX	7 (7.5%)
RCA	10 (10.8%)
LAD+ RCA	4 (4.3%)
LAD+ LCx	1 (1.1%)
LAD+ LCx + RCA	2 (2.2%)
Grafted IMA artery	3 (3.2%)
**SCAD types (*N*** **= 34)** ‡	
Type 1	10 (29.4%)
Type 2	19 (55.9%)
Type 3	2 (5.9%)
Type 1 and 2	1 (2.9%)
Type 2 and 3	1 (2.9%)
Type 1, 2 and 3	1 (2.9%)
**Management**	
PCI	50 (47.6%)
Complicated	6 (12.0%)
Successful	44 (88.0%)
Conservative	49 (46.7%)
DAPT	33 (67.3%)
SAPT	12 (24.5%)
Not specified	4 (8.1%)
CABG	6 (5.7%)
**Outcome**	
In hospital mortality	1 (1.0%)
**Number of recurrences**	
One	15 (14.2%)
Two	1 (1.0%)
Three	1 (1.0%)
Four	1 (1.0%)
**Time of recurrence**	
0–10 days	15 (60.5%)
10 days-6 months	2 (8.3%)
≥6 months	7 (29.2%)

Data are presented as frequency (percentages)

*CCTA* cardiac computed tomography angiography, *IVUS* intravascular ultrasound, *SCAD* spontaneous coronary artery dissection, *LMCA* left main coronary artery, *LAD* left anterior descending artery, *LCx* left circumflex coronary artery, *RCA* right coronary artery, *IMA* internal mammary, *PCI* percutaneous coronary intervention, *CABG* coronary artery bypass graft, *DAPT* dual antiplatelet therapy, *SAPT* single antiplatelet therapy

*ECG evaluation was reported in 96 cases. Data for the remainder (*N* = 9) is unavailable

†Arteries having SCAD were reported in 93 cases. Data for the remainder (*N* = 12) is unavailable

‡SCAD types based on saw classification was reported in 34 cases. Three cases of multivessel SCAD presented with different types of SCAD in each artery

#### ECGs and other clinical diagnoses

Among 96 reported electrocardiograms (ECGs), 7 (7.3%), 34 (35.4%), and 55 (57.3%) patients presented with normal ECGs, ST depression or T segment changes, and ST elevation, respectively. Additionally, 7 (7.3%) patients showed different types of arrhythmias including atrial fibrillation (AF), ventricular fibrillation (VF), or ventricular tachycardia (VT), and a few patients presented with or were diagnosed with cardiogenic shock (*n* = 5) [37, 90, 92, 96, 104], concurrent cardiac tamponade (*n* = 1) [67], and cardiac arrest (*n* = 5) [14, 44, 46, 90, 96].

### Diagnostic imaging modalities

Coronary angiography was used as the first diagnostic modality except for eight patients. Among the rest, cardiac computed tomography angiography **(**CCTA) was either used alone (*n* = 3) [55, 77, 106] or followed by confirmatory angiography (*n* = 5) [32, 43, 51, 54, 70].

In general, 13 patients were initially misdiagnosed by coronary angiography [15, 26, 49, 56, 63, 65, 72, 74, 76, 84, 85, 90, 96] and subsequently secured a diagnosis of SCAD by either intravascular ultrasound (IVUS) (*n* = 2), optical coherence tomography (OCT) (*n* = 3) or repeated angiography (*n* = 8). Among patients with a correct diagnosis by angiography, SCAD was confirmed by IVUS and OCT for 16 and 11 cases, respectively. Additionally, four patients were identified as having simultaneous Takotsubo syndrome [60, 66, 74, 82], and one patient was initially misdiagnosed with Takotsubo syndrome during history taking and physical examination [49].

### Dissected artery characteristics

While the majority of patients had SCAD of a single artery, seven patients had multivessel SCAD [59, 63, 66, 86, 95, 99, 103], with the involvement of four (*n* = 1), three (*n* = 3), and two (*n* = 4) arteries. The most frequently dissected artery was the left anterior descending artery (LAD) (*n* = 70), followed by the right coronary artery (RCA) (*n* = 16), LCx (*n* = 10), and left main coronary artery (LMCA) (*n* = 6). Regarding smaller coronary branches, dissections were found in obtuse marginals (*n* = 6), diagonals (*n* = 3), posterior descending artery (*n* = 3), posterior left ventricular artery (*n* = 2), ramus intermedius (*n* = 1), and atrioventricular circumflex (*n* = 1) branches. Furthermore, three dissections occurred in internal mammary artery grafts among patients with previous coronary artery bypass graft surgery (CABG) [17, 25, 39].

Among 34 patients in whom the SCAD type was reported according to the Saw classification [107], 12 (35.3%) patients were diagnosed with type 1, 22 (64.7%) with type 2, and four (11.8%) with type 3 SCAD. One patient had three arteries having SCAD with three different classification types [86], and two patients had two dissections in two arteries with different types [20, 66]. Initial angiography confirmed type 3 dissection in one patient, which was later found to be type 1 SCAD on repeated angiography [76].

### Management

A total of 49 (46.7%) patients were managed with medical therapy only, 50 (47.6%) with primary percutaneous coronary intervention (PCI), and 6 (5.7%) with CABG. Medical management included the use of either dual (*n* = 33) or single antiplatelet therapy (*n* = 12) in all patients. In addition, other medications, such as statins, beta-blockers, heparin, and warfarin, were frequently used.

Six patients had complicated PCI [14, 19, 52, 85, 90, 103], for whom stenting of the mid-LAD lesion in one patient resulted in a distal expansion of the dissection followed by a retrograde extension to the ostial LAD. A multiple stenting technique was employed to manage distal extensions, and emergent CABG was subsequently performed due to the high risk of LMCA/LCx flow being jeopardized as a result of the proximal extension [85]. PCI was terminated in three patients when the guidewire passage through the false lumen suggested a SCAD diagnosis and medical management was pursued [14, 19, 90]. Similarly, the placement of the guidewire into the false lumen suggested the diagnosis of SCAD in another patient, which was further confirmed using IVUS. Subsequently, the true lumen was found with IVUS guidance, and the stent was deployed at the dissection entry point [52]. Following the stenting of a LAD dissection in another patient, the attempt to stent the RCA lesion was terminated due to difficulties in finding the true lumen and clinical stability following the original PCI [103]. In one of the patients, IVUS imaging showed poor apposition of the stent, and therefore balloon angioplasty was redone [79]. Among the patients managed with CABG, three had LMCA dissection, and one had three arteries with SCAD.

Bioabsorbable vascular stents were utilized in four patients [40, 48, 89, 91]. Intra-aortic balloon pumps were used as additional mechanical support for three patients [90, 102, 104]. Furthermore, three patients participated in cardiac rehabilitation programs, including psychological support [16, 37, 106], and seven patients were advised to abstain from sports [51, 55, 59, 88, 97, 103]..

### Outcome and follow-up events

Among the included cases only 60 cases had a follow up had a follow-up beyond the index hospitalization or initial presentation of SCAD. In-hospital mortality occurred in one of the patients. The patient underwent CABG, and a few hours after the second angiography on the fifth postoperative day, the patient experienced cardiac arrest caused by electromechanical dissociation and died [96]. Two patients experienced VF during their hospital stay and were successfully were defibrillated [15, 92]. During a median follow-up of 2 months, 16 patients experienced SCAD recurrence due to either the progression of a previous lesion or new SCAD in another coronary location, with multiple recurrences in three patients [20, 80, 102]. Just over one-half of patients had recurrence (*n* = 13 (59.1%)) in the first 10 days, with another two (9.1%) experiencing this between 10 days and 6 months, and the rest (*n* = 7 (31.8%)) after 6 months. Eighteen out of 22 recurrences were in individuals who received medical therapy. Univariable analysis of our selected cases revealed that SCAD recurrence was not significantly associated with sex (*p* value = 0.495), hypertension (*p* value = 0.594), FMD (*p* value = 0.261), type of stress (*p* value; physical stress: 0.522, emotional stress: 0.615), or type of management (*p* value; conservative management: 0.138, PCI: 0.141) **(**Table 3**)**. Interestingly, six patients experienced a recurrent episode of chest pain after discharge but were found to be clinically stable in further evaluations [51, 62, 66, 67, 86, 99]. Dual antiplatelet therapy was stopped in one patient due to gastrointestinal bleeding and had recurrent SCAD 8 days following the index event [20].

**Table 3:** Univariable analysis on predictors of SCAD recurrences

Variables		No recurrence *N* (%)	Recurrent *N* (%)	*P* value
**Gender**	Female	50 (57.5%)	11 (61.1%)	0.495
	Male	37 (42.5%)	7 (38.9%)	
**HTN**	No	68 (78.2%)	14 (77.8%)	0.594
	Yes	19 (21.8%)	4 (22.2%)	
**FMD**	No	12 (57.1%)	9 (75.0%)	0.261
	Yes	9 (42.9%)	3 (25.0%)	
**Physical stress**	No	26 (29.9%)	5 (27.8%)	0.552
	Yes	61 (70.1%)	13 (72.2%)	
**Emotional stress**	No	58 (66.7%)	12 (66.7%)	0.615
	Yes	29 (33.3%)	6 (33.3%)	
**Conservative management**	No	49 (56.3%)	7 (38.9%)	0.138
	Yes	38 (43.7%)	11 (61.1%)	
**PCI**	No	43 (49.4%)	12 (66.7%)	0.141
	Yes	44 (50.6%)	6 (33.3%)	
**Arrhythmia**	No	82 (94.3%)	16 (88.9%)	0.344
	Yes	5 (5.7%)	2 (11.1%)	

*FMD* fibromuscular dysplasia, *HTN* hypertension, *PCI* percutaneous coronary intervention

## Discussion

In this systematic review, we studied SCAD patients in whom emotional or physical stress was known as a possible trigger of SCAD. Here, we aimed to compare our findings with the overall data about SCAD from previous studies.

It is hypothesized that a combination of predisposing factors leads to higher susceptibility to having a SCAD event following a trigger. Female sex, pregnancy, physical or emotional stress, and FMD are among the known risk factors proposed by a large number of studies and are more likely to have underlying roles in its pathophysiology. The suggested hypothesis proposes that during physical or emotional stress a sudden catecholamine surge can cause an increase in arterial shear stress and lead to a stress tear of the vasa vasorum [11]. However, this mechanism has not been fully investigated. A similar mechanism has been proposed in other stress-related cardiovascular conditions such as Takotsubo syndrome [11, 108]. Endothelial dysfunction, the initial stage of atherosclerosis, is independently linked to cardiovascular events. Even individuals with few traditional risk factors but with peripheral endothelial dysfunction are at higher risk. Studies have shown mental stress impacts endothelial function, leading to oxidative stress and inflammation, which increase cardiovascular risk [109]. This suggests that the endothelium plays a critical role in translating the physiological effects of mental stress into measurable cardiovascular risk. Additionally, study of Martin et al. [110] showed that individuals with a history of apical ballooning syndrome exhibit abnormal microvascular function when stressed, leading to excessive blood vessel constriction and impaired dilation afterward. Further study revealed impaired responses to acetylcholine in the coronary arteries during mental stress, while responses to nitroglycerin remain intact, indicating endothelial dysfunction [111].

SCAD tends to predominantly affect young or middle-aged women, most often in the peripartum period [2]; surprisingly, in our study, 41.9% of reported stress-related SCAD occurred in men. This discrepancy might be potentially due to the exclusion of pregnancy-associated SCAD in our methods. In our study, the mean age of female patients was significantly higher than that of males likely due in part to excluding women in the peripartum period in the study methodology. However, a study has also reported this age difference [112]. Consistent with the results of other studies [112, 113], we found that SCAD patients whose attacks were precipitated by emotional stress were predominantly women, while physical stressors were reported more frequently in men. The study of Jaskanwal et al. [114] examined a large group of patients with chest pain and nonobstructive CAD. It found that individuals with anxiety disorders, especially women, were more likely to have coronary endothelial dysfunction. This association persisted even after adjusting for traditional cardiovascular risk factors and medication use. The findings suggest that anxiety disorders may contribute to the development of coronary endothelial dysfunction, particularly in women [114]. We witnessed that the presence of coronary artery disease risk factors was consistent with other studies [11, 112, 113, 115–118], wherein hypertension was the most common cardiovascular risk factor reported, and other classic risk factors for MI were not common.

Presently, SCAD patients are routinely screened for FMD due to a strong association, with FMD predicting major adverse cardiovascular events (MACEs) [2, 119]. In the study of Fahmy et al., approximately one-half of the male patients with SCAD had concomitant FMD [112], whereas in our study, all of the FMD cases were women. Takotsubo syndrome and SCAD share key characteristics, prompting questions about a common pathophysiology [120]. Moreover, some articles have proposed a chicken or egg causality between SCAD and Takotsubo syndrome [120]. Recent research indicates that individuals diagnosed with Takotsubo syndrome tend to be older and have a higher prevalence of specific cardiovascular risk factors when compared to those with SCAD. Additionally, Takotsubo syndrome patients, despite their older age and greater cardiovascular risk factors, exhibit lower occurrences of depressive disorder or emotional triggers than SCAD patients [121, 122]. Remarkably individuals with Takotsubo syndrome have a poorer prognosis in terms of in-hospital, mid-term, and long-term outcomes, with higher noncardiac mortality rates compared to SCAD patients [121, 122]. However, it is worth noting that in propensity score-matched cohorts of middle-aged women, SCAD diagnosis resulted in worse long-term outcomes compared to Takotsubo syndrome, primarily due to an elevated risk of cardiac-related rehospitalization [123]. Accordingly, in our study, four patients with stress-related SCAD had concomitant Takotsubo syndrome, and one was initially misdiagnosed with Takotsubo syndrome.

Regarding the clinical manifestations, some studies have reported non-ST elevation myocardial infarction (NSTEMI) as the most common presentation of SCAD [124, 125], but ST elevation myocardial infarction (STEMI) has been more prevalent in SCAD patients in other studies [112, 126]. Similarly, in our selected cases, ST elevation was the most common ECG finding. Additionally, some patients had arrhythmias, cardiogenic shock, cardiac tamponade, and cardiac arrest. These presentations were also reported in previous studies [127].

Coronary angiography has remains the diagnostic gold standard for SCAD [128], yet instances of misdiagnosis (12.4% in this study) emphasize its limitations. Intracoronary imaging (IVUS or OCT) in SCAD poses potential risks, complicating the decision on their application in diagnosis or treatment alongside PCI [129, 130]. Intracoronary imaging can be helpful for diagnostic confirmation of doubtful cases, especially type 3 SCAD [130]. In our study, seven patients had multivessel SCAD. Although one study stated that clinical outcomes and long-term follow-up were similar between single-vessel and multivessel SCAD, the stroke rate was significantly higher in patients with multivessel SCAD [131]. Overall, LAD is reported as the most frequently involved artery in SCAD [8, 125], which was also true in our study. Saw et al. reported type 2 SCAD as the most prevalent angiographic appearance of SCAD [125]. Similarly, the most common type of SCAD in stress-related cases was type 2, accounting for 64.7% of the cases in whom the SCAD type was reported.

Medical management versus revascularization in SCAD patients depends on hemodynamic stability and their thrombolysis in myocardial infarction (TIMI) flow grade found on angiography [132]. Prior meta-analyses indicated that conservative management of SCAD had similar outcomes comparing to the invasive management [133, 134], but in our study, less than one-half of the patients received medical management. It is worth noting that PCI in SCAD patients is technically challenging, given that it can lead to a propagation of dissection and cutoff of coronary flow, putting patients at a higher risk for ischemia and myocardial damage. Despite large cohorts in which SCAD patients are more managed conservatively [119], we witnessed conservative and revascularization strategies used in approximately equal numbers of patients. Therefore, this raises the question of whether this is either a bias of smaller case reports/series or whether patients with a predisposing psychosocial trigger have a slightly more severe phenotype at presentation. Studies have revealed that performing PCI in SCAD patients is challenging, and its success rate is lower than that of PCI in atherosclerotic disease [9, 127].

The incidence of early post discharge readmission following MI and SCAD is considerable. Most readmissions are because of cardiac causes [135]. Recurrence in SCAD occurs more frequently than recurrence in atherosclerotic MI [8, 136]. In our study, 16 patients experienced recurrent SCAD during a median follow-up of 2 months. This observation is consistent with prior literature [137], highlighting the importance of rigorous follow-up in the early post-SCAD period. In a large cohort of SCAD patients with low vascularization rates and high medical management, the recurrence rate was low [119]. However, in our selected SCAD cases, 18 out of 22 recurrences were in individuals who received medical therapy. This discrepancy might be due to the small size of the study population, different demographics, or probably better aggressive treatment results. Investigating the reasons behind this and finding susceptible patients is of utmost importance to reduce the associated morbidity and mortality and prevent unnecessary interventions and hospitalizations. In a meta-analysis by Gerald et al. [138], hypertension and FMD were major stressors for recurrence. In our series, stress-related SCAD recurrences were associated with neither arterial hypertension nor FMD. Additionally, Ehlers–Danlos syndrome, ADPKD, and Loeys–Dietz syndrome were present in patients with SCAD recurrence.

## Limitations

There were some limitations in our study. To evaluate detailed information of each individual, we included only case reports and case series. We excluded other observational studies due to insufficient data on each individual, although they had larger populations. This is considered as the major limitation of the present study. Additionally, it should be noted that having heterogeneous follow-up durations could impact data consistency and analysis in our study and we were unable to provide a complete and comprehensive evaluation of follow-up events.

## Conclusion

**Fig. 2:**
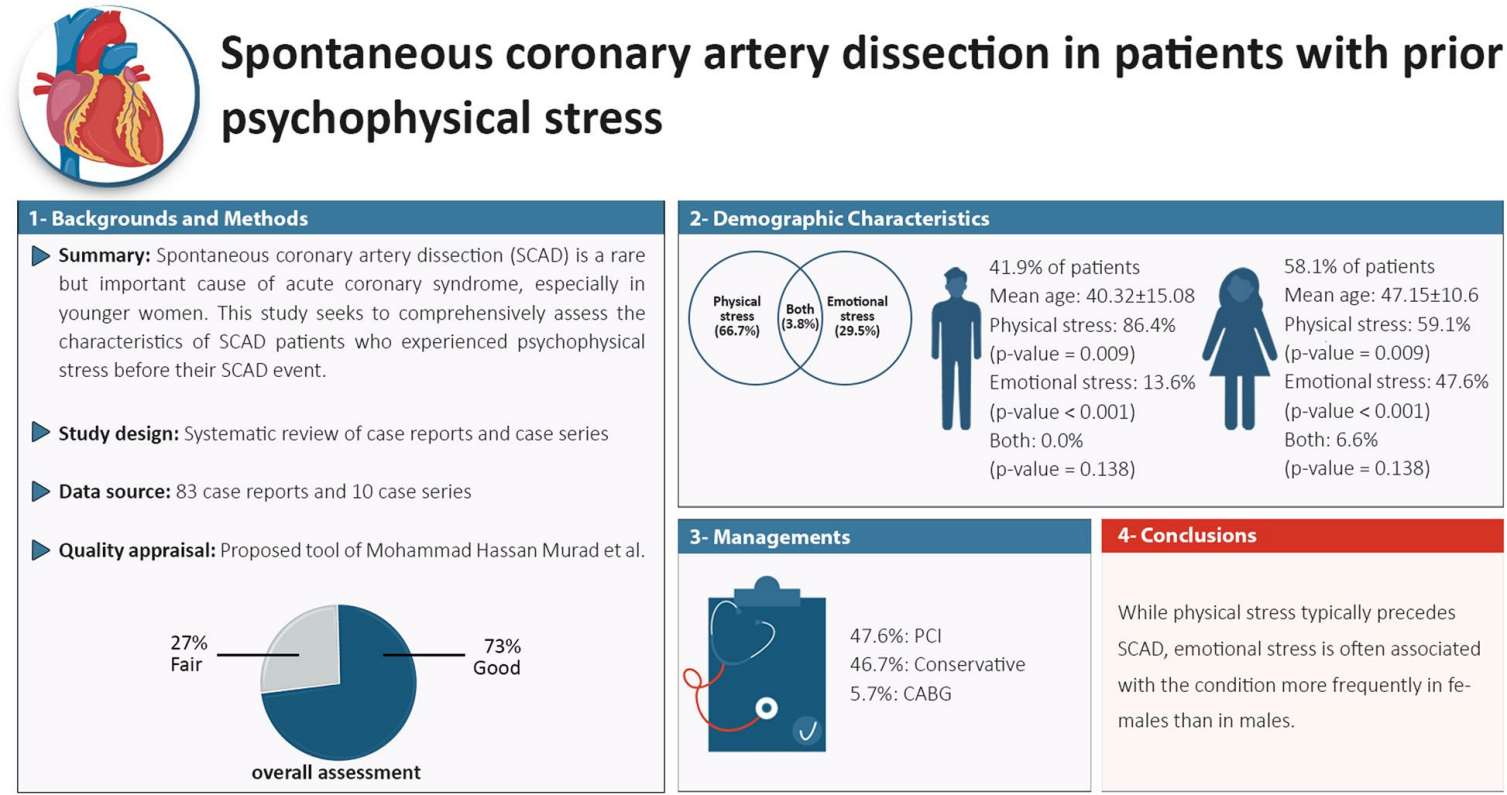
Graphical abstract

In the present systematic review of case reports and case series, we described the stress type, clinical features, and angiographic findings of SCAD in patients with prior psychophysical stress (see Fig. 2). While physical stress seems to precede SCAD in most cases, emotional stress is implicated in females more than males. Further studies are needed to confirm these findings.

### Supplementary Information

**Supplementary Material 1.**

**Supplementary Material 2.**

## Data Availability

The dataset supporting the conclusions of this article is included within the article and its additional files.
