# Supplementary material for: Spontaneous coronary artery dissection in patients with prior psychophysical stress: a systematic review of case reports and case series
Source: BMC Cardiovasc Disord. 2024 May 3;24:235. doi: 10.1186/s12872-024-03902-2 (PMC11067298; doi:10.1186/s12872-024-03902-2)
Supplement: Supplementary file 1 — Supplementary Material 1. [file 12872_2024_3902_MOESM1_ESM.docx]

The methodological quality of the case reports and case series was evaluated using six of the eight questions recommended in the referenced article (**Supplementary Table 1)**.

**Supplementary Table 1. Quality appraisal**

| Author, Year | 1 | 2 | 3 | 4 | 5 | 6 | 7 |
| --- | --- | --- | --- | --- | --- | --- | --- |
| Aksakal, 2014 | Y | Y | Y | Y | Y | Y | Good |
| Almahmeed, 1995 | Y | Y | Y | Y | Can’t tell | Y | Good |
| Altshuler,2021 | Y | Y | Y | N | Y | N | Fair |
| Alvarez, 2019 | Y | Y | Y | Y | Y | Y | Good |
| Anderson, 2015 | Y | Y | Y | N | Y | Y | Fair |
| Anuwatworn, 2017 | Y | Y | Y | Y | N | N | Fair |
| Aqel, 2004 | Y | Y | Y | N | Y | Y | Good |
| Arrivi, 2012 | Y | Y | Y | Y | Y | Y | Good |
| Asrar ul Haq, 2013 | Y | Y | Y | Y | Y | Y | Good |
| Azam Khan, 2006 | Y | Y | N | Y | Y | Y | Good |
| Bath, 2019 | Y | Y | Y | Y | Y | Y | Good |
| Ben Ahmed, 2020 | Y | Y | Y | Y | Y | Y | Good |
| Bethan Nia Thomas, 2014 | Y | Y | Y | Y | N | Y | Good |
| Capuano, 2006 | Y | Y | Y | Y | Y | Y | Good |
| Cerrato, 2015 | Y | Y | Y | Y | Y | Y | Good |
| Chaaban, 2022 | Y | Y | Y | Y | Y | Y | Good |
| Chang, 2017 | Y | Y | Y | Y | N | N | Fair |
| Cockburn, 2014 | Y | Y | N | Y | Y | Y | Good |
| Costa Cascais, 2021 | Y | Y | Y | Y | Y | Y | Good |
| Cropp, 2013 | Y | Y | N | Y | N | Y | Fair |
| De souza, 2019 | N | Y | Y | Y | Y | Y | Good |
| DeBoer, 2022 | N | Y | Y | Y | Y | Y | Good |
| Di Marco, 2016 | Y | Y | Y | Y | Y | Y | Good |
| Dong Ho, 2007 | Y | Y | N | Y | N | Y | Fair |
| Dwyer, 2006 | Y | Y | Y | Y | Y | Y | Good |
| Ellis, 1994 | Y | Y | Y | N | Y | Y | Good |
| Emren, 2015 | Y | Y | Y | Y | N | Y | Good |
| Eugene, 2015 | Y | Y | Y | Y | Y | Y | Good |
| Farouji, 2021 | Y | N | Y | Y | N | Y | Fair |
| Fitouchi, 2022 | Y | Y | Y | Y | Y | Y | Good |
| Fitzpatrick, 2020 | Y | Y | Y | Y | Y | Y | Good |
| Ghafoor, 2020 | Y | Y | Y | Y | Y | Y | Good |
| Goh, 2015 | Y | Y | Y | Y | Y | N | Good |
| Guess, 2018 | Y | Y | Y | Y | Y | Y | Good |
| H Lin, 2012 | Y | Y | Y | Y | Y | Y | Good |
| Hassan, 2017 | Y | Y | N | Y | Y | N | Fair |
| Igleasias, 2011 | Y | Y | N | Y | Y | N | Fair |
| Jacqueline Saw, 2011 | Y | Y | N | Y | N | Y | Fair |
| Jatti, 2021 | Y | Y | Y | Y | Y | Y | Good |
| Joy, 2019 | Y | Y | N | Y | N | Y | Fair |
| Kalaga, 2007 | Y | Y | Y | Y | N | Y | Good |
| Kalinskaya, 2019 | Y | Y | Y | Y | Y | Y | Good |
| Kanaroglou, 2015 | Y | N | Y | Y | Can’t tell | Y | Fair |
| Kang, 2020 | Y | Y | Y | Y | Y | Y | Good |
| Karabag, 2011 | Y | Y | N | Y | N | Y | Fair |
| Karl Poon, 2010 | Y | N | Y | Y | N | Y | Fair |
| Kegai, 2021 | Y | Y | Y | Y | N | Y | Good |
| Khan, 2022 | Y | Y | Y | Y | Y | Y | Good |
| Kireev, 2020 | Y | Y | Y | Y | N | Y | Good |
| Lempereur,2014 | Y | N | Y | Y | N | Y | Fair |
| Maeder, 2004 | N | Y | Y | Y | Y | Y | Good |
| Mahendiran, 2022 | Y | Y | Y | Y | Can’t tell | Y | Good |
| Maiga, 2022 | Y | N | Y | Y | N | Y | Fair |
| Mayr,2010 | Y | Y | N | Y | Y | N | Fair |
| Mehrani, 2018 | Y | Y | Y | Y | Y | Y | Good |
| Miki, 2012 | Y | Y | Y | Y | N | Y | Good |
| Mohammadian, 2022 | Y | Y | Y | Y | N | Y | Good |
| Muzaffar Mahmood,2016 | Y | Y | Y | N | Y | Y | Good |
| Nadeem Jafri, 2018 | Y | Y | Y | Y | N | Y | Good |
| Oluwole,2020 | Y | Y | Y | Y | Y | Y | Good |
| Onoda,2018 | Y | Y | Y | Y | Y | Y | Good |
| Papanikolaou, 2020 | Y | N | Y | Y | Y | N | Fair |
| Parry, 1994 | Y | N | Y | Y | N | Y | Fair |
| Phogat, 2022 | Y | Y | Y | Y | Can’t tell | Y | Good |
| Rahman, 2021 | Y | Y | Y | Y | Y | Y | Good |
| Riaz, 2020 | Y | Y | Y | Y | Y | Y | Good |
| Roche, 2022 | Y | Y | Y | Y | Can’t tell | Y | Good |
| Sawami, 2019 | Y | Y | Y | Y | N | Y | Good |
| Sengottuvelu, 2014 | Y | N | Y | Y | Y | N | Fair |
| Sharma,2019 | Y | Y | Y | Y | Y | Y | Good |
| Sharma, 2016 | Y | N | Y | Y | Y | N | Fair |
| Shenoy, 2020 | Y | Y | Y | Y | Y | Y | Good |
| Singh, 2014 | Y | Y | Y | Y | Y | Y | Good |
| Sivam, 2013 | Y | Y | Y | Y | N | N | Fair |
| Smith, 2018 | Y | N | Y | Y | N | Y | Fair |
| Suresh,2007 | Y | N | Y | Y | Y | N | Fair |
| Sutil-Vega, 2019 | Y | N | Y | Y | Y | Y | Good |
| Tagliari,2017 | Y | Y | Y | Y | Y | Y | Good |
| Taha, 2018 | Y | Y | Y | Y | Can’t tell | Y | Good |
| Tanabe, 2021 | Y | Y | Y | Y | N | Y | Good |
| Unnikrishnan, 2018 | Y | Y | Y | Y | N | Y | Good |
| V. Sherrid, 1995 | Y | Y | Y | Y | Y | N | Good |
| Vale, 1998 | Y | Y | Y | Y | Y | Y | Good |
| Vandamme, 2016 | Y | Y | Y | Y | Y | Y | Good |
| Vandeloo, 2018 | Y | Y | N | Y | N | Y | Good |
| Verlaeckt, 2019 | Y | Y | Y | Y | Y | Y | Good |
| Afzal, 2015 | Y | Y | Y | Y | Y | Y | Good |
| Wong, 2004 | Y | Y | Y | Y | N | Y | Good |
| Xin-He, 2013 | Y | N | Y | Y | Y | Y | Good |
| Yang, 2018 | Y | Y | Y | Y | N | Y | Good |
| Yeung, 2019 | Y | Y | Y | Y | N | Y | Good |
| Yiangou, 2016 | Y | Y | N | Y | N | Y | Fair |
| Yierong, 2018 | Y | Y | Y | Y | Y | Y | Good |

1: Does the patient(s) represent(s) the whole experience of the investigator (Centre) or is the selection method unclear to the extent that other patients with similar presentation may not have been reported?

2: Was the exposure adequately ascertained?

3: Was the outcome adequately ascertained?

4: Were other alternative causes that may explain the observation ruled out?

5: Was follow-up long enough for outcomes to occur?

6: Is the case(s) described with sufficient details to allow other investigators to replicate the research or to allow practitioners make inferences related to their own practice?

7: Quality rating (Good≥5, Fair=4, Poor≤3)
