# Supplementary material for: Spontaneous coronary artery dissection in patients with prior psychophysical stress: a systematic review of case reports and case series
Source: BMC Cardiovasc Disord. 2024 May 3;24:235. doi: 10.1186/s12872-024-03902-2 (PMC11067298; doi:10.1186/s12872-024-03902-2)
Supplement: Supplementary file 2 — Supplementary Material 2. [file 12872_2024_3902_MOESM2_ESM.docx]

Additional documented comorbidities and data for each patient individually are presented in **Supplementary Table 2**.

**Supplementary Table 2. Included studies characteristics**

| **Author, study year** | **Study design** | **Region** | **Age** | **Gender** | **Type of stress** | **Stress definition** | **Dissected coronary artery** | **Dissection type** |
| --- | --- | --- | --- | --- | --- | --- | --- | --- |
| Roche (1), 2022 | case-report | AMR | 54 | F | Emotional | She had recently learned that she and several family members were facing imminent unemployment, reportedly due to COVID-19 vaccine refusal. | LAD | NA |
| Mohammadian (2), 2022 | case-report | AMR | 46 | M | Physical | He was in the gym and was lifting weights. | LAD | 2 |
| Phogat (3), 2022 | case-report | AMR | 60 | F | Physical | Working out at the gym | NA | NA |
| Mahendiran (4), 2022 | case-report | EUR | 65 | F | Physical | Scuba diving | NA | NA |
| Maiga (5), 2022 | case-report | EUR | 60 | F | Emotional | Funeral of her mother | LAD | 3 |
| DeBoer (6), 2022 | case series | EUR | 22 | F | Emotional | Not specified | LAD | 2 |
| Chaaban (7), 2022 | case-report | AMR | 37 | F | Physical | Having dinner | LAD | 2 |
| DeBoer (6), 2022 | case series | EUR | 39 | F | Emotional | Significant emotional stress related to family and work matters | LAD | 2 |
| Khan (8), 2022 | case-report | AMR | 47 | F | Emotional | Significant emotional trauma | LAD | 2 |
| Fitouchi (9), 2022 | case-report | EUR | 49 | F | Emotional | Very intense stress disorder | LAD | 2 |
| Costa Cascais (10), 2021 | case report | EUR | 17 | M | Physical | Push ups | LAD | NA |
| Jatti (11), 2021 | case-report | EUR | 39 | M | Emotional | Post-assault stress | LMCA | NA |
| Rahman (12), 2021 | case-report | AMR | 44 | M | Physical | Moved heavy furniture around the household | LAD | NA |
| Tanabe (13), 2021 | case-report | WPR | 52 | F | Emotional | Father’s funeral | LAD | 2 |
| Kegai (14), 2021 | case-report | WPR | 68 | F | Physical and emotional | Had been caring for her grandchildren for a few days | LAD | 2 |
| Farouji (15), 2021 | case-report | AMR | 41 | M | Physical | Vigorous sexual intercourse | LAD | 2 |
| Altshuler (16), 2021 | case-report | AMR | 58 | M | Physical | Lifting machinery | LAD | 2 |
| Ghafoor (17), 2020 | case-report | AMR | 52 | F | Emotional | Funeral of her mother | LAD, RCA | 2, 3 |
| Fitzpatrick (18), 2020 | case-report | EUR | 59 | M | Physical | 5km-run, morning run | NA | 2 |
| Ben Ahmed (19), 2020 | case report | AFR | 46 | F | Emotional | Lost her father two days ago | LAD, LCx, RCA | 1, 2, 3 |
| Kang (20), 2020 | case-report | AMR | 39 | F | Physical | Aerobic exercise | LAD | NA |
| Riaz (21), 2020 | case-report | AMR | 45 | F | Emotional | Acute distress | LAD | NA |
| Papanikolaou (22), 2020 | case-report | EMR | 51 | F | Physical | Intense cough | LAD | NA |
| Shenoy (23), 2020 | case-report | AMR | 19 | M | Physical | Running a 5km race | LAD | NA |
| Kireev (24), 2020 | case-report | EUR | 35 | M | Physical | Gardening | NA | NA |
| Oluwole (25), 2020 | case-report | AMR | 26 | F | Physical | Exercising on the treadmill | NA | 2 |
| Yeung (26), 2019 | case-report | AMR | 54 | F | Physical and emotional | Bowel movement,,mother's funeral | NA | 1, 2 |
| Sutil-Vega (27), 2019 | case-report | EUR | 54 | F | Physical | Training at the gym | LCx | NA |
| Kalinskaya (28), 2019 | case-report | EUR | 47 | F | Emotional | Air travel caused emotional stress. | LAD | 1 |
| Sawami (29), 2019 | case-report | WPR | 78 | F | Emotional | Grandchild's death | LCx | NA |
| De souza (30), 2019 | case series | AMR | 20 | M | Physical | 30 min to 1h of  high-intensity exercise | RCA | NA |
| Verlaeckt (31), 2019 | case-report | EUR | 44 | F | Emotional | - | LAD | 1 |
| Joy (32), 2019 | case-report | EUR | 48 | F | Physical and emotional | Yoga | LAD | NA |
| Sharma (33), 2019 | case-report | EUR | 53 | F | Emotional | Bereavement of a friend | RCA | NA |
| De souza (30), 2019 | case series | AMR | 29 | M | Physical | Related to  intense efforts (soccer training) | LAD | NA |
| De souza (30), 2019 | case series | AMR | 31 | M | Physical | Started after  practicing 1h of football. | RCA | NA |
| Bath (34), 2019 | case-report | AMR | 48 | M | Physical | Mowing grass | LAD | NA |
| Alvarez (35), 2019 | case-report | EUR | 53 | F | Physical | Exertion | NA | 1 |
| Nadeem Jafri (36), 2018 | case-report | AMR | 36 | M | Physical | Cycling and bicycle fall | LAD | 1 |
| Unnikrishnan (37), 2018 | case-report | AMR | 22 | M | Physical | Playing basketball | LMCA | NA |
| Vandeloo (38), 2018 | case-report | EUR | 52 | M | Physical | Lifting heavy weights | LAD | NA |
| Mehrani (39), 2018 | case-report | EMR | 33 | M | Physical | From 2 days ago he began ‘light body building and aerobic exercise’. Retrosternal chest discomfort was associated with holding a 30 pounds weight at the first time. | LCx | NA |
| Guess (40), 2018 | case-report | AMR | 26 | M | Physical | 15 minutes after completing a military physical fitness test | LAD | 1 |
| Smith (41), 2018 | case-report | AMR | 45 | F | Physical and emotional | Shortly after a meeting with her husband to  discuss their divorce proceedings. She recently started  a high-intensity interval training program, 1.5 weeks prior to presentation | NA | NA |
| Yang (42), 2018 | case-report | WPR | 49 | F | Physical | Intense physical activity | LAD | NA |
| Weber (43), 2018 | case-report | AMR | 22 | M | Physical | Cycling | LAD | NA |
| Yierong (44), 2018 | case-report | AMR | 55 | M | Physical | Violent cough episodes | NA | NA |
| Onoda (45), 2018 | case-report | WPR | 55 | M | Physical | Playing golf | RCA | NA |
| Taha (46), 2018 | case-report | AMR | 43 | M | Physical | Jiu-jitsu class | LAD | 2 |
| Tagliari (47), 2017 | case-report | AMR | 48 | M | Physical | Physical exertion | LAD | NA |
| Hassan (48), 2017 | case-report | EUR | 54 | F | Emotional | The patient had lost her husband 6 months prior to her index presentation and has experienced episodes of panic disorder since then. | RCA | NA |
| Anuwatworn (49), 2017 | case-report | AMR | 36 | F | Physical | Exertion | LCx | NA |
| Chang (50), 2017 | case-report | WPR | 25 | M | Physical | Lifting a heavy object | LAD | NA |
| Vandamme (51), 2016 | case series | EUR | 51 | F | Emotional | Discussion with her husband. | LAD | NA |
| Vandamme (51), 2016 | case series | EUR | 28 | M | Physical | Strenous physical exercise | LAD, RCA | NA |
| Di Marco (52), 2016 | case-report | EUR | 42 | F | Emotional | Intense psychological stress | LAD | NA |
| Muzaffar Mahmood (53), 2016 | case-report | EUR | 42 | F | Physical | She had been carrying chairs at her place of work prior to the onset of severe chest pain. | LAD | NA |
| Sharma (54), 2016 | case series | SEAR | 19 | M | Physical | Cricket match | LAD | NA |
| Yiangou (55), 2016 | case-report | AMR | 54 | F | Physical | Gardening and disposing of a heavy basket of cut grass | LAD | 1 |
| Goh (56), 2015 | case-report | AMR | 70 | F | Physical | Lifting her husband | LAD | NA |
| Kanaroglou (57), 2015 | case-report | AMR | 47 | M | Physical | Gardening for up to 4 h | NA | NA |
| Andreson (58), 2015 | case series | WPR | 52 | F | Emotional | - | LAD, RCA | 2 |
| Eugene (59), 2015 | case-report | EUR | 48 | F | Emotional | Got fired from her job | LAD | NA |
| Cerrato (60), 2015 | case-report | EUR | 48 | F | Physical | Trekking excursion over mountains | LAD | NA |
| Andreson (58), 2015 | case series | WPR | 55 | F | Emotional | - | NA | NA |
| Emren (61), 2015 | case-report | EUR | 32 | M | Emotional | Verbal disscussion in a family environment | LAD | 2 |
| Lempereur (62), 2014 | case-report | AMR | 54 | F | Emotional | Very stressful work week of litigation | LAD | 2 |
| Aksakal (63), 2014 | case-report | EUR | 70 | M | Emotional | Relative's funeral | LAD, LCx | 1 |
| Singh (64), 2014 | case-report | AMR | 34 | F | Emotional | Arguing with another parent at her daughter’s soccer match | LAD | NA |
| Sengottuvelu (65), 2014 | case-report | SEAR | 42 | F | Physical | Yoga | LMCA | NA |
| Cockburn (66), 2014 | case-report | WPR | 43 | M | Physical | Bench pressing heavy weights | NA | NA |
| Bethan Nia Thomas (67), 2014 | case series | EUR | 49 | M | Physical | Lifting of heavy machinery at work | LAD | NA |
|  |  |  | 53 | M | Emotional | He described recent anxiety and emotional distress associated with a highly stressful and demanding job, and had been feeling particularly anxious around the time of the initial chest pain. | LCx | NA |
|  |  |  | 40 | M | Physical | Intense exercise | LAD | 3 |
| Xin-He (68), 2013 | case series | WPR | 50 | F | Physical | During farm work | RCA | 2 |
| Asrar ul Haq (69), 2013 | case-report | WPR | 54 | M | Physical | Heavy swimming 3 h prior to presentation | LAD, RCA | 1 |
| Cropp (70), 2013 | case-report | AMR | 14 | F | Physical | Collapsed during the second lap of a race | LMCA | NA |
| Sivam (71), 2013 | case-report | WPR | 45 | F | Physical | Cough | LAD | NA |
| Arrivi (72), 2012 | case-report | EUR | 38 | F | Emotional | Intense period of psychological stress (she recently applied for a divorce). | LAD | NA |
| H Lin (73), 2012 | case-report | AMR | 43 | F | Physical | After valsalva manoeuvre during a bowel movement | LAD | NA |
| Miki (74), 2012 | case-report | WPR | 50 | F | Physical | Bowel movement | RCA | 1 |
| Igleasias (75), 2011 | case-report | EUR | 42 | F | Emotional | Not specified | LAD | 2 |
| Jacqueline Saw (76), 2011 | case series | AMR | 48 | F | Physical | 2 days after running 10 km. | LAD | NA |
|  |  |  | 35 | F | Physical | Aerobic exercises. | RCA | NA |
|  |  |  | 42 | F | Physical | 30 minutes after intercourse | LAD | NA |
|  |  |  | 45 | F | Physical | A day after skiing | LAD | NA |
|  |  |  | 48 | F | Physical | After lifting her child | LAD | NA |
| Karabag (77), 2011 | case-report | EUR | 35 | M | Emotional | - | LAD, RCA, LCx | 1 |
| Mayr (78), 2010 | case-report | EUR | 51 | F | Emotional | Massive emotional pressure at the office | LAD | NA |
| Karl Poon (79), 2010 | case-report | AMR | 39 | F | Physical | Rigorous aerobic exercises. | LAD | NA |
| Suresh (80), 2007 | case-report | SEAR | 75 | M | Physical | Lifting a heavy bookcase | LAD | NA |
| Dong Ho (81), 2007 | case-report | WPR | 46 | F | Physical | Lifting furniture | LAD | NA |
| Kalaga (82), 2007 | case-report | AMR | 17 | M | Physical | Started during a pick-up basketball game | LAD | NA |
| Azam khan (83), 2006 | case series | AMR | 52 | F | Physical | Washing dishes | RCA | NA |
| Dwyer (84), 2006 | case-report | WPR | 21 | M | Emotional | Exciting final minutes of a football match | LAD | NA |
| Capuano (85), 2006 | case-report | EUR | 56 | M | Physical | Exertional exercise | LAD | NA |
| Wong (86), 2004 | case-report | AMR | 69 | M | Physical | Digging up a post box | LAD | NA |
| Aqel (87), 2004 | case-report | EUR | 52 | M | Physical | Loading bales of hay to his truck | LAD | NA |
| Maeder (88), 2004 | case series | EUR | 49 | F | Physical | 30 km inline-skating trip | LCx | NA |
|  |  |  | 53 | F | Physical | Doing housework | LAD | NA |
| Vale (89), 1998 | case-report | WPR | 28 | M | Physical | Baseball match | LCx | NA |
| Almahmeed (90), 1995 | case-report | AMR | 32 | F | Physical | During an aerobics class | LMCA | NA |
| V. Sherrid (91), 1995 | case-report | AMR | 41 | M | Physical | Running | LAD | NA |
| Ellis (92), 1994 | case-report | EUR | 35 | F | Physical | Severe exercise | LMCA | NA |
| Parry (93), 1994 | case-report | EUR | 42 | M | Physical | Vigorous training program | RCA | 2 |

AMR, region of the Americas; SEAR, south east Asia region; EUR, European region; EMR, eastern Mediterranean region; F,female; LAD, leftt anterior descending cornary artery; LCx, left circumflex artery; LMCA, left main coronary artery; RCA, right coronary artery; NA, not available; SEAR, south east Asia region; WPR, western Pacific region

**References**

1. Roche AM, Klingel K, Toth K, Pepper K, Francis SA. Spontaneous Coronary Artery Dissection in the Setting of COVID-19 Pandemic-Related Stressors: A Case Report. Cureus. 2022;14(3):e23069.

2. Mohammadian M, Shah D, Santana M, Elkattawy S, Jesani S. Levine's Sign Points to Spontaneous Coronary Artery Dissection in a Healthy Young Male. Cureus. 2022;14(5):e24893.

3. Phogat V, Nepal S, Kozman H. A Non-atherosclerotic Heart Tears Apart: A Case of Spontaneous Coronary Artery Dissection in a Healthy Postmenopausal Woman. Cureus. 2022;14(5):e25459.

4. Mahendiran T, Desgraz B, Antiochos P, Rubimbura V. Case Report: A First Case of Spontaneous Coronary Artery Dissection Potentially Associated With Scuba Diving. Front Cardiovasc Med. 2022;9:855449.

5. Maiga H, Poropat Flerin T, Berlot B, Lipar L, Cvijic M. Challenging diagnosis of spontaneous coronary artery dissection: how to look beyond the obvious. Eur Heart J Case Rep. 2022;6(11):ytac422.

6. DeBoer R, Nasir U, Fraga JD. Spontaneous Coronary Artery Dissection: Chest Pain in a Young Woman. J Community Hosp Intern Med Perspect. 2022;12(4):70-4.

7. Chaaban N, Kshatriya S. Spontaneous Coronary Artery Dissection With Systemic Lupus Erythematosus. Ochsner J. 2022;22(4):353-5.

8. Khan H, Yousaf A, Ahmad M, Munir A, Moza A. Complete Recovery of an Occluded Coronary Artery Secondary to Spontaneous Coronary Artery Dissection With Medical Management in a Young Patient Presenting With Acute Coronary Syndrome. Cureus. 2022;14(10):e29980.

9. Fitouchi S, Di Marco P, Motreff P, Lhoest N. Concomitant presentation of spontaneous coronary artery dissection with Takotsubo syndrome: a case report. Eur Heart J Case Rep. 2022;6(5):ytac172.

10. Costa Cascais F, Pereira AR, Almeida AR, Rocha L. A rare cause of chest pain in paediatric age: a teenager with acute myocardial infarction due to spontaneous coronary artery dissection. BMJ Case Rep. 2022;15(1).

11. Jatti K, Maurovich-Horvat P, Hasleton J, Uddin M, Ruzsics B. Cardiac Computed Tomography to Identify and Guide Therapy of Intramural Hemorrhage in High-Risk Coronary Anatomy. JACC Case Rep. 2021;3(1):120-4.

12. Rahman T, Moghadam R, Rinder M. Spontaneous Coronary Artery Dissection: An Unusual Cause of ST-Elevation Myocardial Infarction in Young Males. Cureus. 2021;13(1):e12827.

13. Junya T, Yuzo K, Akihiro E, Kazuaki T. Spontaneous coronary artery dissection associated with psychological stress. BMJ Case Reports. 2021;14(8):e245414.

14. Kegai S, Sato K, Goto K, Ozawa T, Kimura T, Kobayashi K, et al. Coexistence of Spontaneous Coronary Artery Dissection, Takotsubo Cardiomyopathy, and Myocardial Bridge. JACC: Case Reports. 2021;3(2):250-4.

15. Farouji I, Al-Radideh O, Abed H, DaCosta TR, Battah A, Ahmad AS, et al. Unusual Presentation of Post-coital Spontaneous Coronary Artery Dissection. Cureus. 2021;13(8):e17460.

16. Altshuler E, Matthia E, Naik D, Keeley EC. Extremely Heavy Lifting Associated With Spontaneous Coronary Artery Dissection. Cureus. 2021;13(11):e19451.

17. Ghafoor HU, Bose A, El-Meligy A, Hannan J. A case report of recurrent spontaneous coronary artery dissection and Takotsubo cardiomyopathy: a treatment dilemma. Eur Heart J Case Rep. 2020;4(1):1-6.

18. Fitzpatrick JJ, Noman A, Ryan N, Dawson DK. Recurrent spontaneous coronary artery dissection in a middle-aged male athlete patient: a case report. Eur Heart J Case Rep. 2020;4(4):1-5.

19. Ben Ahmed H, Allouche E, Rekik A, Ouechtati W, Bezdah L. Multivessel spontaneous coronary artery dissection with simultaneously three different angiographic patterns. Ann Cardiol Angeiol (Paris). 2022;71(2):118-21.

20. Kang G, Sarraju A, Nishi T, Rogers I, Tremmel JA, Kim JB. Spontaneous Coronary Artery Dissection and ST-Segment Elevation Myocardial Infarction in an Anomalous LAD Artery. JACC Case Rep. 2020;2(1):45-50.

21. Riaz S, Vasigh M, Mogadam E, Ganesan D, Chaudhuri D. Acute Myocardial Infarction Due to Spontaneous Coronary Artery Dissection and Plaque Rupture. Cureus. 2020;12(5):e8063.

22. Papanikolaou J, Alharthy A, Platogiannis N, Balhamar A, Alqahtani SA, Memish ZA, et al. Spontaneous coronary artery dissection in a patient with COVID-19. Coronary Artery Disease. 2021;32(4):354-5.

23. Shenoy P, Tayeb T, Covas P, Temesgen N, Tracy C. Not Your Common Athletic Heart Problem: Using Coronary CTA to Visualize Spontaneous Coronary Artery Dissection. Case Rep Cardiol. 2020;2020:8882561.

24. Kireev K, Genkel V, Kuznetsova A, Sadykov R. Multivessel spontaneous coronary artery dissection in a patient after mild COVID-19: A case report. SAGE Open Med Case Rep. 2020;8:2050313x20975989.

25. Oluwole AS, Virk HUH, Witzke C. Intense exercise may not be so benign: chest pain in a young athletic woman may be spontaneous coronary artery dissection (SCAD). BMJ Case Rep. 2020;13(4).

26. Yeung DF, Saw J. Multiple recurrences of spontaneous coronary artery dissection in a woman with fibromuscular dysplasia. Catheter Cardiovasc Interv. 2019;94(5):702-5.

27. Sutil-Vega M, Romeu Vilar D, Barros-Membrilla AJ, Millán X, Hidalgo JA, Pons-Lladó G. Case 270: Spontaneous Coronary Artery Dissection Associated with Fibromuscular Dysplasia. Radiology. 2019;293(1):235-40.

28. Kalinskaya A, Skrypnik D, Kostin A, Vasilieva E, Shpektor A. Case Report of an Acute Myocardial Infarction as a Result of Spontaneous Coronary Artery Dissection in a Patient with Fibromuscular Dysplasia. Case Rep Cardiol. 2019;2019:3051616.

29. Sawami K, Natsuaki M, Hongo H, Kajiwara M, Kaneko T, Inoue Y, et al. Spontaneous Internal Mammary Artery Graft Dissection Triggered by Emotional Stress. JACC Case Rep. 2019;1(5):732-6.

30. Souza P, Herdy AH. Spontaneous Exercise-Related Coronary Artery Dissection among Young Patients Without Risk Factors or Atherosclerotic Disease. Arq Bras Cardiol. 2019;113(5):988-98.

31. Verlaeckt E, Van de Bruaene L, Coeman M, Gevaert S. Spontaneous coronary artery dissection in a patient with hereditary polycystic kidney disease and a recent liver transplant: a case report. Eur Heart J Case Rep. 2019;3(4):1-5.

32. Joy G, Eissa H. Spontaneous Coronary Artery Dissection in a Patient with a Family History of Fatal Ascending Aortic Dissection: Case Report and Discussion of Diseases Causing Both Presentations. Case Rep Cardiol. 2019;2019:7218480.

33. Sharma H, Vetrugno V, Khan SQ. Successful treatment of a spontaneous right coronary artery dissection with a 4-mm diameter cutting balloon: a case report. Eur Heart J Case Rep. 2019;3(4):1-6.

34. Bath AS, Aggarwal S, Gupta V, Kalavakunta JK. Slit in the Coronaries: A Case of Spontaneous Coronary Artery Dissection. Cureus. 2019;11(6):e4841.

35. Álvarez-Lario B, Álvarez-Roy L, Mayordomo-Gómez S, García-García JM. Spontaneous coronary artery dissection in systemic lupus erythematosus: case-based review. Rheumatol Int. 2019;39(10):1821-7.

36. Jafri FN, Solarz D, Hjemdahl-Monsen C. Cycling Induced Spontaneous Coronary Artery Dissection in a Healthy Male. Case Rep Emerg Med. 2018;2018:2740513.

37. Unnikrishnan D, Annam R, Jacob A, Thyagarajan B, Farrugia P. STEMI in a Young Male after Use of Synephrine-Containing Dietary Supplement. Case Rep Cardiol. 2018;2018:7074104.

38. Vandeloo B, Azzano A, Schoors D, Verstraeten A, Van Laer L, Loeys B, et al. Spontaneous Coronary Artery Dissection in a Man With a Novel Missense Mutation in SMAD2 Treated by Optical Coherence Tomography-Guided Percutaneous Coronary Intervention. JACC Cardiovasc Interv. 2019;12(6):e45-e7.

39. Mehrani M, Nematollahi A, Hatami M, Hosseini K. Coronary artery dissection in a 33-year-old man with fatigue and episodic retrosternal burning: a case report. European Heart Journal - Case Reports. 2018;2(3):yty068.

40. Guess JM, Madigan CG, Hudspath CB, Hurley JT, Martinho S. Spontaneous Coronary Artery Dissection in a 26-Year-Old-Male Soldier. Military Medicine. 2019;184(5-6):e462-e6.

41. Smith AAH, Wananu M, Carlson MD. Spontaneous Coronary Artery Dissection in a Healthy Woman after Initiating a High-Intensity Interval Training Workout Program. Am J Cardiol. 2018;122(9):1588-9.

42. Yang XQ, Zhu HY, Wang X, Zhao HB, Zhang W, Xiao M, et al. Spontaneous coronary artery dissection in a middle-aged woman with acute anterior myocardial infarction: A case report. Medicine (Baltimore). 2018;97(31):e11504.

43. Weber N, Weber A, Carbone P, Lawrence A, Bilbrey T, Schussler JM, et al. High-intensity, sport-specific cardiac rehabilitation training of a 22-year-old competitive cyclist after spontaneous coronary artery dissection. Proc (Bayl Univ Med Cent). 2018;31(2):207-9.

44. Yirerong JA, Hurlburt H. Spontaneous coronary artery dissection in a man with intense coughing spasms. BMJ Case Rep. 2018;2018.

45. Onoda N, Izumi K. A case of spontaneous coronary artery dissection complicated with pseudoaneurysm healed by medical treatment: Follow-up by multidetector computed tomography. J Cardiol Cases. 2018;18(5):156-9.

46. Taha M, Latt H, Al-Khafaji J, Ali M, Seher R. A case of spontaneous coronary artery dissection presenting with acute anterior wall myocardial infarction in a young adult male - an increasingly recognized rare disease. J Community Hosp Intern Med Perspect. 2018;8(2):60-3.

47. Tagliari AP, Kochi AN, Rohde LEP, Wender OCB. Spontaneous Left Anterior Descending Coronary Artery Dissection Requiring Coronary Artery Bypass Surgery. Braz J Cardiovasc Surg. 2017;32(6):536-8.

48. Y-Hassan S, Themudo R, Maret E. Spontaneous coronary artery dissection and takotsubo syndrome: The chicken or the egg causality dilemma. Catheterization and Cardiovascular Interventions. 2017;89(7):1215-8.

49. Anuwatworn A, Sethi P, Steffen K, Jonsson O, Petrasko M. Spontaneous Coronary Artery Dissection: A Rare Manifestation of Alport Syndrome. Case Rep Cardiol. 2017;2017:1705927.

50. Chang FL, Chang WC, Cheng YT, Liu TJ, Lee WL, Lai CH. Spontaneous coronary artery dissection causing acute myocardial infarction and cardiac arrest in a 25-year-old male. Perfusion. 2018;33(2):160-3.

51. Vandamme M, De Backer J, De Backer T, Drieghe B, Devos D, Gevaert S. The spectrum of spontaneous coronary artery dissection: illustrated review of the literature. Acta Cardiol. 2017;72(6):599-609.

52. Di Marco M, Clemente D, Forlani D, D'Alleva A, Duronio G, Paloscia L. Spontaneous coronary artery dissection in a young woman resolved with conservative strategy. A case report. J Cardiol Cases. 2016;14(2):59-61.

53. Mahmood MM, Austin D. IVUS and OCT guided primary percutaneous coronary intervention for spontaneous coronary artery dissection with bioresorbable vascular scaffolds. Cardiovasc Revasc Med. 2017;18(1):53-7.

54. Sharma S, Raut N, Potdar A. Spontaneous coronary artery dissection: Case series and review of literature. Indian Heart J. 2016;68(4):480-5.

55. Yiangou K, Papadopoulos K, Azina C. Heavy Lifting Causing Spontaneous Coronary Artery Dissection with Anterior Myocardial Infarction in a 54-Year-Old Woman. Tex Heart Inst J. 2016;43(2):189-91.

56. Goh AC, Lundstrom RJ. Spontaneous Coronary Artery Dissection with Cardiac Tamponade. Tex Heart Inst J. 2015;42(5):479-82.

57. Kanaroglou S, Nair V, Fernandes JR. Sudden cardiac death due to coronary artery dissection as a complication of cardiac sarcoidosis. Cardiovasc Pathol. 2015;24(4):244-6.

58. Anderson RD, Jayadeva PS, Wilson WM, Iyer R. Spontaneous Coronary Artery Dissection: Case Series from a Tertiary Centre. Heart Lung Circ. 2016;25(3):e41-5.

59. Eugène M, Siam-Tsieu V, Pillière R, Deblaise J, Dubourg O, Mansencal N. Recurrent spontaneous coronary artery dissection: Unexpected evolution and major role of emotional stress. Int J Cardiol. 2015;201:316-8.

60. Cerrato E, Tomassini F, Rolfo C, Gagnor A, Varbella F. Spontaneous coronary artery dissection treated with biovascular scaffolds guided by intravascular ultrasounds imaging. Cardiovasc Interv Ther. 2017;32(2):186-9.

61. Emren SV, Şenöz O, Duygu H, Nazlı C, Ergene O. Primary spontaneous coronary dissectİon in a young male and the role of intravascular ultrasonography for diagnosis and treatment. International Journal of the Cardiovascular Academy. 2015;1(2):66-8.

62. Lempereur M, Gin K, Saw J. Multivessel spontaneous coronary artery dissection mimicking atherosclerosis. JACC Cardiovasc Interv. 2014;7(7):e87-8.

63. Aksakal A, Arslan U, Yaman M, Urumdaş M, Ateş AH. Spontaneous coronary artery dissection as a cause of myocardial infarction. World J Cardiol. 2014;6(12):1290-2.

64. Singh GD, Nishimura M, Rogers JH, Amsterdam EA. Pain at the game: spontaneous coronary artery dissection. Am J Med. 2014;127(12):1160-3.

65. Sengottovelu G, Rajendran R, Dattagupta A. Optical coherence tomographic image of dynamic left main coronary artery compression caused by intramural haematoma due to spontaneous coronary artery dissection – degloved artery managed with bioresorbable vascular scaffold. EuroIntervention. 2015;11(6):659.

66. Cockburn J, Yan W, Bhindi R, Hansen P. Spontaneous coronary artery dissection treated with bioresorbable vascular scaffolds guided by optical coherence tomography. Can J Cardiol. 2014;30(11):1461.e1-3.

67. Thomas BN, Aslam S, Cullen J, Anantharaman R. Spontaneous coronary artery dissection in men presenting with acute coronary syndrome, successfully managed by intravascular ultrasound-guided percutaneous coronary intervention. BMJ Case Rep. 2014;2014.

68. Xin-He Y, Cheng-Jian Y, Yan J, Xin X, Jia-Ning C, Zhen-Jie Y, et al. A successful emergency management of spontaneous coronary artery dissection and review of the literature. Am J Emerg Med. 2013;31(7):1156.e1-3.

69. Asrar Ul Haq M, Mutha V, van Gaal WJ. Multivessel spontaneous coronary artery dissection of left and right coronary systems. BMJ Case Rep. 2013;2013.

70. Cropp EM, Turner JS, Kreutz RP. Spontaneous coronary artery dissection in a 14-year-old. Am J Emerg Med. 2013;31(2):461.e5-7.

71. Sivam S, Yozghatlian V, Dentice R, McGrady M, Moriarty C, Di Michiel J, et al. Spontaneous coronary artery dissection associated with coughing. J Cyst Fibros. 2014;13(2):235-7.

72. Arrivi A, Milici C, Bock C, Placanica A, Boschetti E, Dominici M. Idiopathic, serial coronary vessels dissection in a young woman with psychological stress: a case report and review of the literature. Case Rep Vasc Med. 2012;2012:498465.

73. Lin AH, Shutt BJ, Dendall RT, Bennett W. Multivessel spontaneous coronary artery dissection treated with staged percutanous coronary intervention in a non-postpartum female. BMJ Case Rep. 2012;2012.

74. Miki K, Fujii K, Nakata T, Shibuya M, Fukunaga M, Kawai K, et al. The utility of intravascular ultrasound for the diagnosis and management of spontaneous coronary artery dissection in a middle-aged woman with acute inferior myocardial infarction. J Cardiol Cases. 2012;6(3):e78-e80.

75. Iglesias D, Salinas P, Jiménez-Valero S. Spontaneous coronary artery dissection evaluated by optical coherence tomography. Journal of Cardiovascular Medicine. 2011;12(10).

76. Saw J, Poulter R, Fung A, Wood D, Hamburger J, Buller CE. Spontaneous Coronary Artery Dissection in Patients With Fibromuscular Dysplasia. Circulation: Cardiovascular Interventions. 2012;5(1):134-7.

77. Karabag T, Dogan SM. A case of spontaneous multivessel coronary artery dissection presenting with acute myocardial infarction and ventricular tachycardia. Catheterization and Cardiovascular Interventions. 2012;79(1):113-6.

78. Mayr A, Klug G, Jaschke W, Pachinger O, Metzler B. Persistent spontaneous dissection of the left anterior descending coronary artery after emotional pressure. Wien Klin Wochenschr. 2010;122(15-16):515-7.

79. Poon K, Bell B, Raffel OC, Walters DL, Jang IK. Spontaneous coronary artery dissection: utility of intravascular ultrasound and optical coherence tomography during percutaneous coronary intervention. Circ Cardiovasc Interv. 2011;4(2):e5-7.

80. Suresh V, Evans S. Successful stenting of stenotic lesion and spontaneous dissection of left internal mammary artery graft. Heart. 2007;93(1):44.

81. Ho YD, Koizumi T, Lee DP. Spontaneous coronary artery dissection in a woman with depression without coronary atherosclerotic risk factors. J Invasive Cardiol. 2007;19(6):E166-8.

82. Kalaga RV, Malik A, Thompson PD. Exercise-related spontaneous coronary artery dissection: case report and literature review. Med Sci Sports Exerc. 2007;39(8):1218-20.

83. Khan NU, Miller MJ, Babb JD, Ahmed S, Saha PK, Shammas RL, et al. Spontaneous coronary artery dissection. Acute Card Care. 2006;8(3):162-71.

84. Dwyer N, Galligan L, Harle R. Spontaneous coronary artery dissection and associated CT coronary angiographic findings: a case report and review. Heart Lung Circ. 2007;16(2):127-30.

85. Capuano C, Sesana M, Predolini S, Leonzi O, Cuccia C. Case report: a very large dissection in the left anterior descending coronary artery of a 56-year-old man. Cardiovasc Revasc Med. 2006;7(4):240-2.

86. Wong P, Rubenstein M, Inglessis I, Pomerantsev E, Ferrell M, Leinbach R. Spontaneous spiral dissection of a LIMA-LAD bypass graft: a case report. J Interv Cardiol. 2004;17(4):211-3.

87. Aqel RA, Zoghbi GJ, Iskandrian AE. Spontaneous coronary artery dissection with pseudoaneurysm formation diagnosed by intravascular ultrasound: a case report. Echocardiography. 2004;21(2):153-7.

88. Maeder M, Ammann P, Angehrn W, Rickli H. Idiopathic spontaneous coronary artery dissection: incidence, diagnosis and treatment. Int J Cardiol. 2005;101(3):363-9.

89. Vale PR, Baron DW. Coronary artery stenting for spontaneous coronary artery dissection: a case report and review of the literature. Cathet Cardiovasc Diagn. 1998;45(3):280-6.

90. Almahmeed WA, Haykowski M, Boone J, Ling H, Allard M, Webb J, et al. Spontaneous coronary artery dissection in young women. Catheterization and Cardiovascular Diagnosis. 1996;37(2):201-5.

91. Sherrid MV, Mieres J, Mogtader A, Menezes N, Steinberg G. Onset during exercise of spontaneous coronary artery dissection and sudden death. Occurrence in a trained athlete: case report and review of prior cases. Chest. 1995;108(1):284-7.

92. Ellis CJ, Haywood GA, Monro JL. Spontaneous coronary artery dissection in a young woman resulting from an intense gymnasium "work-out". Int J Cardiol. 1994;47(2):193-4.

93. Parry R, MacConnell T, Wilde P. Case report: spontaneous coronary artery dissection. Clin Radiol. 1994;49(2):142-3.

* Other documented comorbidities included asthma, migraine headache, cystic fibrosis, chronic obstructive pulmonary disease, COVID-19 infection, toxic thyroid goiter, Alport syndrome, systemic lupus erythematosus, Loeys‒Dietz syndrome, Ehlers‒Danlos syndrome, autosomal dominant polycystic kidney disease (ADPKD), and non-Hodgkin lymphoma.
